# Supplementary material for: Risk factors associated with cardiovascular disease among adult Nevadans
Source: PLoS One. 2021 Feb 17;16(2):e0247105. doi: 10.1371/journal.pone.0247105 (PMC7888645; doi:10.1371/journal.pone.0247105)
Supplement: S1 Table — (DOCX) [file pone.0247105.s001.docx]

| **S1 Table. Sociodemographic Factors Associated with CVD by Year** | | | | |
| --- | --- | --- | --- | --- |
| **Calculated Variable** | **2011** | | **2017** | |
|  | **OR** | **95% CI** | **OR** | **95% CI** |
| **Gender** |  |  |  |  |
| Female * | 1.00 | (1.00, 1.00) | 1.00 | (1.00, 1.00) |
| Male | **1.64** | **(1.15, 2.33)** | **1.92** | **(1.29, 2.85)** |
| **Income** |  |  |  |  |
| < $15,000 | **2.25** | **(1.34, 3.77)** | **2.37** | **(1.20, 4.65)** |
| $15,000 — $25,000 | **2.06** | **(1.19, 3.56)** | 1.64 | (0.92, 2.93) |
| $25,000 — $35,000 | 1.48 | (0.76, 2.88) | **2.30** | **(1.19, 4.42)** |
| $35,000 — $50,000 | 0.91 | (0.50, 1.63) | 1.07 | (0.62, 1.87) |
| > $50,000 * | 1.00 | (1.00, 1.00) | 1.00 | (1.00, 1.00) |
| **Age** |  |  |  |  |
| 18-64 | **0.17** | **(0.12, 0.25)** | **0.27** | **(0.18, 0.40)** |
| 65 or older * | 1.00 | (1.00, 1.00) | 1.00 | (1.00, 1.00) |
| **Race** |  |  |  |  |
| White, non-Hispanic * | 1.00 | (1.00, 1.00) | 1.00 | (1.00, 1.00) |
| Black, non-Hispanic | **2.01** | **(1.12, 3.61)** | 0.70 | (0.31, 1.56) |
| Hispanic | 0.82 | (0.40, 1.68) | 0.57 | (0.31, 1.03) |
| Other Race | 0.71 | (0.39, 1.32) | 0.96 | (0.40, 2.30) |
| *Reference group | | | | |
