# Supplementary material for: Risk factors associated with cardiovascular disease among adult Nevadans
Source: PLoS One. 2021 Feb 17;16(2):e0247105. doi: 10.1371/journal.pone.0247105 (PMC7888645; doi:10.1371/journal.pone.0247105)
Supplement: Attachment — Submitted filename: Response to Reviewers Table 1.28.21.docx [file pone.0247105.s002.docx]

**Manuscript ID PONE-D-20-26195 for PLOS ONE Response to Reviewers Revisions/Responses**

Risk Factors Associated with Cardiovascular Disease among Adult Nevadans

| **Reviewers’ Comments** | **Authors’ Revisions/Responses** |
| --- | --- |
| **Reviewer #1**  The purpose of this cross-sectional study is to examine the association between self-reported risk factors (heavy drinking, smoking, high cholesterol, hypertension, overweight/obesity, and diabetes) and CVD presence among adult Nevadans. However, some issues need to be clarified or revised:  1) Some of the self-reported risk factors (cholesterol, hypertension, diabetes) are not behavioral risk factors. The title of this manuscript is improper.  2) Several variables such as hypertension, diabetes, high cholesterol, obesity (BMI) and smoking are not clearly defined. Please give details.  3) The description of methodology and data analysis is not rigorous enough. For example, the raking weighting methodology should be more specific including the weighted variables. The BRFSS Data User Guide of CDC should also be referenced.  4) Please show the data in line122-134 in a table format.  5) 95% confidence intervals (CIs) and the missing number of each subgroup are necessary in Table1, 2 and 3.  6) In order to avoid ambiguity, weighted percentages should also be illustrated below each table.  7) Some data in Table 2 and 3 do not have percentages. (e.g., 9--,23--,20--, etc) Please illustrate the meaning below each table. Meanwhile, please add the abbreviations.  8) There are several data errors in these tables. Please recheck them carefully. (e.g., the percentage of female current smoker in Table 3 is15.8%; number of male CVD in Table 3 is 164, etc)  9) Nineteen references may be insufficient. Further discussion is needed instead of retelling the results.  10) Please do not use semicolons in sentences！  11) Several minor errors should be revised as follow: “mg/dL” should be “mg/d” in line 41; some brackets are italic; “0.23 0.08, 0.62)” should be “0.23 (0.08, 0.62)” in line 139.  **Reviewer #2**  1. The study examined the association between risk factors and CVD presence among adult Nevadans between 2011 and 2017, but it is less objective since the study relied on the self-reporting.  2. Multiple logistic regressions were performed to examine the association of different demographic and risk factors with CVD presence in Nevada by year. Please add a table showing all the result, so difference would be obvious between 2011 and 2017.  3. Age was categorized into four groups: 18-24 years old, 25-44 years old, 45-64 years old, and 65 years and 100 older. Could you please give a reason of age categories in this study.  4.BMI (overweight and obese) remained the most prevalent CVD risk factor. However, compared to normal weight, there was huge difference of underweight individuals on reporting CVD(Table 5). Please provide some further discussion on it. | **Reviewer #1**      1. Thank you for pointing this out. We have edited the title to remove “behavioral.” Our current title is *Risk Factors Associated with Cardiovascular Disease among Adult Nevadans.*  2. We have created a table that provided the operational definition for all these variables, Table 1. We have edited the tables to follow the appropriate order.  3. We have edited the methodology section to expand on our descriptions and we have including the BRFSS Data User Guide reference, which it a part of reference 9, the CDC BRFSS website.  4. We have the tables with the multiple logistic regressions examining the association of different demographic and risk factors with CVD; however, we only included two out of the three tables due to the table limits. We included in this revision that the editor can have as a supplementary document, Supplementary Table 1.  5. Thank you for your suggestions; unfortunately, adding the 95% Cl would not fit easily in tables 2 and 3 (edited to be only one table 3). It will be too cluttered for the tables. We provided the p-value to simplify the table. Age and gender differences among different risk factors were analyzed by chi-square. The missing number are unsure and no response, so not enough data to report percentage.  6. The information provided in each table is in weighted percentages.  7. Please refer to response #5. We have added the abbreviations for Table 3.  8. We apologize for the oversight. We have reviewed and edited the tables appropriately.  9. We expanded our discussion section and the number of references has been increased to reflect these edits.  10. We apologize for the excessive use of semicolons. We have edited the manuscript appropriately to avoid using semicolons except before the usage of “therefore” and “however.”  11. We apologize for the oversight. Thank you for pointing these out, we have edited the errors.  **Reviewer #2**  1. Thank you for taking the time to review our manuscript. We do agree that self-reporting is not the ideal methodology which we discussed under the limitations. Despite the limitations, we do find that this manuscript have merits to contribute to the body of knowledge. BRFSS dataset has been wisely used to answer research questions in an inexpensive manner. BRFSS dataset also have a large sample size that have better representation of the states and national data. Moreover, self-reports of nationally representative sample are found to as useful as objective measures.  2. Please refer to response #4 from Reviewer 1.  3. We tried to capture the different age groups of adulthood (early adulthood, young adult, middle age, and older adults) for the ease of data comparison to other publish data, especially national data.  4. We have expanded our explanation on BMI toward the end of the discussion. |
